# Anti-*Helicobacter pylori*, anti-Inflammatory, and Antioxidant Activities of Trunk Bark of *Alstonia boonei* (Apocynaceae)

**DOI:** 10.1155/2022/9022135

**Published:** 2022-09-15

**Authors:** Zenab Linda Fagni Njoya, Marius Mbiantcha, Stephanie Flore Djuichou Nguemnang, Vanessa Mba Matah Marthe, William Yousseu Nana, Yacine Karelle Madjo Kouam, Elvira Ngoufack Azanze, Eric Gonzal Tsafack, Gilbert Ateufack

**Affiliations:** ^1^Laboratory of Animal Physiology and Phytopharmacology, Faculty of Science, University of Dschang, Cameroon; ^2^Laboratory of Biology and Physiology of Animal Organisms, Department of Biology of Animal Organisms, Faculty of Science, The University of Douala Cameroon, P.O. Box 24157, Douala, Cameroon

## Abstract

An ulcer is an erosion of the gastric mucosa that occurs following an imbalance between the aggression and protective factors and/or an infection with *Helicobacter pylori* (*H. pylori*). About 90-100% of duodenal ulcers and 70-80% of gastric ulcers are caused by *H. pylori*. The objective of this work was to evaluate in *vitro* the anti-*H. pylori* activity and then the anti-inflammatory and antioxidant properties of aqueous and methanol extracts of *Alstonia boonei*. The anti-*H. pylori* tests (CMI and antiureasic activity) were determined using the agar well diffusion method, the microbroth dilution method, and the measurement of ammonia production by the indophenol method; the anti-inflammatory properties were evaluated by inhibition of proteinases, denaturation of albumin, production of NO by macrophages, cell viability, and hemolysis of red blood cells by heat; then, the antioxidant properties were evaluated by the FRAP method (ferric reducing antioxidant power) and the DPPH (1,1-diphenyl-2-picrylhydrazyl) test. The results show that the best trapping of the DPPH radical was obtained with the methanol extract (EC_50_ = 8.91 *μ*g/mL) compared to the aqueous extract (EC_50_ = 19.86 *μ*g/mL). The methanol extract also showed greater iron-reducing activity than the aqueous extract and vitamin C. Furthermore, at the concentration of 200 *μ*g/mL, the methanol extract showed a percentage (96.34%) strains of *H. pylori* higher than that of the aqueous extract (88.52%). The MIC_90_ of the methanol extract was lower than that of the aqueous extract. The methanol extract showed a higher percentage inhibition (85%) of urease than the aqueous extract (73%). The methanol extract at a concentration of 1000 *μ*g/mL showed the greatest ability to inhibit proteinase activity, albumin denaturation, and red blood cell hemolysis; on the other hand, maximum cell viability and greater production of nitrite oxide by macrophages were obtained with the aqueous extract. Aqueous and methanol extracts of *Alstonia boonei* possess anti-*H. pylori* which would probably be linked to their antioxidant and anti-inflammatory properties.

## 1. Introduction

*Helicobacter pylori* (*H. pylori*), spiral bacterium, which colonizes the gastric mucosa of living things of about half of the world's population, first begins in the gastric mucosa during infancy and persists asymptomatically throughout of life in the absence of effective treatment [1, 2]. Its prevalence varies from country to country, and this bacterium affects more than 50% of people worldwide. Infection rates are higher in areas such as Africa, Eastern Europe, Central America, and Central Asia [3]. A study by Hooi et al. [4] had a high rate in Africa with a prevalence of 70.1%; in Cameroon, the prevalence of this infection is 54.3% in the city of Douala [5] and 43.4% in the health district of Melong [6]. It is known that the risk of developing gastric carcinoma and/or gastric cancer is greatly increased in an individual infected with the *H. pylori* bacterium [7]. Moreover, the presence of this bacterium in the stomach can alter the histology of the mucosa, leading to chronic gastritis, intestinal metastasis, atrophy, dysplasia, and ultimately gastric cancer [8, 9]. Other disorders such as esophageal adenocarcinoma, multiple sclerosis, gastroesophageal reflux disease, inflammatory bowel disease, and asthma may occur after *H. pylori* infection; as well as peptic ulcers in 75% of cases [10, 11].

Chronic *H. pylori* infection is able to promote inflammation that triggers apoptosis of stomach epithelial cells accompanied by an accumulation of epigenetic changes leading to alterations in signaling pathways, which results in a disruption cell differentiation, epithelial cell renewal, and gastric epithelial homeostasis [8, 12]. Since the low pH of the gastric lumen is an important factor in limiting bacterial growth, one of the methods used by *H. pylori* to colonize, persist, and survive in the conditions of low gastric pH is the increase in pH urease activity. This enzyme hydrolyses urea generates ammonia which will buffer the cytoplasm, the periplasm, and the direct environment of the *H. pylori* bacterium [13, 14]. Furthermore, mutant strains of *H. pylori* lacking urease have been shown to be unable to colonize persistently the gastric mucosa, justifying the importance of this enzyme for *H. pylori* infection [15]. *H. pylori* infection induces inflammation accompanied by overproduction of reactive oxygen and nitrogen species with consequent loss of cell homeostasis and cell death [12], which leads to a deregulation of the signaling pathways that promote the survival and proliferation of gastric epithelial cells in a harmful environment that will later promote the development of cancer [16]. The inflammation of the gastric mucosa (gastritis) due to *H. pylori* is the consequence of the stimulation of the production of proinflammatory cytokines, and chemokines, which will lead to the recruitment of numerous immune cells (macrophages, neutrophils, and lymphocytes) which will contribute to mucosal damage [2, 17–19]. During *H. pylori* infection, many microbial pathogens cause oxidative stress in infected cells [20, 21], leading to extensive epithelial damage [22]. Furthermore, oxidative stress plays a role in altered epithelial proliferation, increased oxidative DNA damage [23, 24], and increased apoptosis associated with infection with *H. pylori* [24]. In addition, *H. pylori* infection increases the expression and activity of spermine oxidase, which has the ability to oxidize polyamines in epithelial cells releasing hydrogen peroxide [25]. There is also a family of proteases in the stomach which plays an important role in bacterial species, located in the periplasmic space, and they contribute to the virulence of microorganisms after their secretion, because they participate in the export of virulence factors and their maturation. *H. pylori* proteases include components of tight junctions, adhesion junctions, and extracellular matrix proteins that facilitate the breakdown of proteolytic substrates and the gastric epithelial cell layer [26].

When gastritis and/or duodenitis persists, there is release and activation of leukocytes which lead to hypersensitivity of other inflammatory cells causing tissue damage in the mucosa; the secretion of gastric acid leads to the severity of these lesions, hence the appearance of gastric and/or duodenal ulcers. Generally, the combination of a proton pump inhibitor, clarithromycin, and amoxicillin or metronidazole (for patients allergic to penicillin) for seven to 14 days represents the first-line treatment to eradicate *H. pylori* [27]. However, it is known that the use of proton pump inhibitors can increase the risk of enteric infections such as Campylobacter and Salmonella, community-acquired pneumonia [28], and Clostridium difficile infections [29] and spontaneous bacterial peritonitis [30] and that *H. pylori* is extremely resistant to many antibiotics, and these reasons make it difficult to treat *H. pylori* infection. On the other hand, other treatments such as anticholinergics, antimicrobial agents, antacids, sucralfate, H_2_ receptor antagonists, and bismuth are ineffective but also produce many adverse effects such as impotence hematopoietic alterations, arrhythmia, gynecomastia, and hypersensitivity [31, 32]. For these reasons, many gastroprotective drugs have emerged from pharmacological research on medicinal plants. In particular, plants with antioxidant capacity as the main mechanism are used as a reservoir for the treatment of ulcer disease [32]. Many studies have been done to discover potential anti-*H. pylori*, anti-inflammatory, and antioxidant in herbal medicine. The manufacture as well as the pharmacological and/or clinical evaluation of medicinal products derived from plants has made it possible to transform natural (herbal) medicine into a modern society that can make important contributions to the delivery of health care [33]. In addition, medicinal plants are the main source of new drugs for the prevention or treatment of gastric ulcer [34].

The Apocynaceae represents a vast plant family comprising 5,000 species and 350 genera. It includes lianas, herbaceous plants, trees, and shrubs. *Alstonia boonei* (*A. boonei*) belongs to this vast family of plants with many therapeutic virtues. It is a deciduous tree whose bark and roots are used in Nigeria as an antimalarial [35], in Burkina and Côte d'Ivoire, and a decoction of the bark is used to clean the wounds [36]. In India and Ghana, the bark is used to relieve pain and to treat rheumatoid arthritis. In Cameroon, the bark of *A. boonei* is used against chronic diarrhea and ulcerative colitis [37]. In addition, *A. boonei* has shown its antioxidant [38], antimicrobial [39], and anticolitis [37] properties. In addition, the aqueous extract of *A. boonei* possesses antiulcerogenic and antiulcer properties and exhibits low toxicity [40]. The objective of this study was to evaluate in vitro the anti-*H. pylori* and then the anti-inflammatory and antioxidant properties of aqueous and methanol extracts of *A. boonei*.

## 2. Material and Methods

### 2.1. Collection and Extraction of the Plant

The trunk bark of *A. boonei* was collected in April 2019, in the west region of Cameroon (Foumban). After authentication of the plant (M. Nana Victor, Botanist) at the National Herbarium of Cameroon (Yaoundé) by comparison (specimen N°43368/HNC), the bark was harvested, cleaned, cut into small pieces, dried (in the shade), and ground to obtain a powder, which was used for the preparation of the various extracts. For the aqueous extract, 450 g of this powder was soaked in 4.5 liters of distilled water and boiled (100°C, 15 minutes); after cooling, the mixture was filtered (Wattman N° 1 paper); then, the filtrate was dried (oven, 40°C, 3 days) to give 22 g of extract (4.89% yield). For the methanol extract, 250 g of the powder was soaked in methanol (3 liters); then, the mixture was macerated (48 hours, room temperature); then, the mixture was filtered; and the filtrate was passed through Buchi Rotavapor (R-124, 65°C) under reduced pressure to give 12.68 g of extract (5.07% yield).

### 2.2. Phytochemical Tests of *A. boonei* Extracts

#### 2.2.1. Qualitative Phytochemical

Qualitative phytochemistry of the aqueous and methanol extract of *A. boonei* was performed to determine the presence of certain bioactive compounds. Thus, several tests were carried out, including the test for alkaloids, flavonoids, tannins, polyphenols, triterpenes, sterols, saponins, glucosides, anthocyanins, and anthraquinones. Flavonoids, tannins, polyphenols, and saponin tests were carried out according to the protocol described by Harbone [41], while the test for glucosides, alkaloids, anthocyanins, and anthraquinones was done as described by Odebeyi and Sofowara [42]. Triterpenes and sterols were qualified by the methods described by Trease and Evans [43] and Sofowara [42], respectively.

#### 2.2.2. Quantitative Phytochemical

The total amount of phenols present within the extracts was determined using Folin-Ciocalteu's reagent. In carrying out this study, gallic acid was used as our standard alongside total phenols which are expressed as mg/g of gallic acid equivalents (GAE). 0.01, 0.02, 0.03, 0.04, and 0.05 mg/mL were concentrated with gallic acid and prepared using methanol. After preparation, concentrations of 0.1 and 1 mg/mL of extract were also prepared using methanol. 0.5 mL from each of the samples was placed in test tubes and mixed with 2.5 mL of a Folin-Ciocalteu reagent which was diluted 10 times alongside 2 mL of a solution of 7.5 mg of gallic acid and 2 mL of 7.5% sodium carbonate. All the tubes were covered and placed on a stand for 30 minutes at room temperature, after which the absorbance was read using a spectrophotometer at 760 nm. As the Folin-Ciocalteu reagent is sensitive to reducing compounds, including polyphenols, it produced blue color during the reaction [44].

The totality of flavonoid content gotten from the extracts was estimated using the method proposed by Zhishen et al. [45]. Each 1 mL sample was mixed with 4 mL of distilled water and 0.3 mL of NaNO_2_ solution (10%). Five minutes later, 0.3 mL of AlCl_3_ solution (10%) was included into the mixture followed by 2 mL of NaOH solution (1%). Absorbance was ascertained at 510 nm relative to blank.

The tannin content gotten from each sample was obtained using insoluble polyvinylpolypyrrolidone (PVPP), which binds tannins as described by Makkar et al. [46]. One (1) mL extract of *A. boonei* was dissolved in 1 mg/mL of methanol, and total phenols were determined and mixed with a 100 mg of PVPP, vortexed for a period of 15 minutes at 4°C, and later centrifuged for 10 minutes at 3.000 rpm. The clear supernatant, nontannin phenols were determined in the same way as total phenols; the tannin content was calculated as the difference between total phenols and nontannin phenol content.

### 2.3. Evaluation of anti-*H. pylori* Activity of *Alstonia boonei* Extracts

#### 2.3.1. Bacterial Strains

Fifteen resistant strains of *H. pylori* were isolated from gastric biopsies gotten from patients suffering from gastric ulcer infections despite treatment with metronidazole and amoxicillin and undergoing endoscopy at the Dr. Panjwani Center for Molecular Medicine and Drug Research, International Center for Chemical and Biological Sciences. The human biopsy specimens used in this study were received from a donor in accordance with the procedure accepted by the Independent Ethics Committee (International Center for Chemical and Biological Sciences (ICCBS)), University of Karachi, No: ICCBS/IEC-015-BC-2019/Protocol/2.1. A standard control strain (NCTC 11638) was also included. The biopsies were homogenized in aseptic conditions of 0.2 g/L cysteine and 20% glycerol in brain heart infusion (BHI) broth (Oxoid, England). A loopful of homogenate was spread on a base of freshly prepared Columbia agar (Oxoid, England), supplemented with 6% of horse blood and Skirrow's supplement (Oxoid, England) containing trimethoprim (2.5 mg), vancomycin (5 mg), cefsulodin (2.5 mg), and amphotericin (2.5 mg), and finally, the inoculated plates were incubated at 37°C for a period of five days under microaerophilic conditions (5-6% O_2_, 10% CO_2_, and 80-85% N_2_) (Anaerocult Basingstoke, Hampshire, England). Isolates were identified on the basis of colony morphology, positive oxidase, urease and catalase tests, and glmM gene amplification. Confirmed isolates was suspended in Eppendorf tubes containing 1 mL of BHI broth and 20% glycerol, stored at -80°C for further use. Gastric biopsies were obtained from consenting patients who were not on antibiotics, such as PPIs, or bismuth salts for duration of at least one week.

#### 2.3.2. Evaluation of Aqueous and Methanol Extracts of *A. boonei* on Strains of *H. pylori*

The agar diffusion method described by Boyanova et al. [47] was used for this test. *H. pylori* inocula were prepared with McFarland turbidity standard 2 and placed on BHI agar supplemented with 5% of horse blood and Skirrow's supplements. The inocula were distributed evenly on a plate, after which the plate containing the inocula was dried for 15 minutes. Six millimeter (6 mm) diameter wells were dilled into the agar making using a sterile stainless steel drill, then filled with 65 *μ*L of each extract at a concentration of 100 mg/mL. Sixty-five (65) *μ*L of 0.05 *μ*g/mL clarithromycin and 10% DMSO (dimethylsulfoxide) were included in all experiments as a positive and negative control, respectively. The plates were incubated under microaerophilic conditions at 37°C for a duration of 72 hours, after which the diameters of the zones of inhibition were measured in mm. The experiment was performed in duplicate, and the middle areas were recorded. A zone diameter ≥ 14 mm was used as the sensitivity breaking point for clarithromycin and for the extracts, which was also used in calculating the sensitivity percentage [48]. A plate inoculated with a reference of strain (NCTC 11638) of *H. pylori* was included in all experimental series.

#### 2.3.3. The Minimum Inhibitory Concentration (MIC_90_)

More than 50% of strains were sensitive to aqueous extracts and methanol. They were therefore chosen for determining the minimum inhibitory concentration (MIC) using the method of dilution in micro-plates, following the method of Bonacorsi et al. [49]. The test was performed using 96-well plates. Extracts were prepared at a concentration of 5.0 mg/mL and filtered using a 2.0-*μ*m filter. Double dilution of the extracts was made in BHI broth supplemented with 5% of horse serum and Skirrow supplements. Final concentration of each extract was between 0.001 and 5.0 mg/mL, and twenty *μ*L of 18 hours *H. pylori* broth culture suspension (McFarland turbidity standard 2) was added to 100 *μ*L of culture medium containing each extract. Control wells were prepared with culture medium plus bacterial suspension including broth, respectively. Metronidazole and amoxicillin were used as standard drugs, at concentrations ranging from 0.005 to 5.0 mg/mL and 0.001 to 1.25 mg/mL. The plates were incubated at 37°C for a duration of 72 hours under microaerophilic conditions, and absorbance was read at 620 nm using an automatic enzyme-linked immuno assay (ELISA) microplate reader (Tokyo, Japan). The initial and post-incubation absorbances were compared in order to detect an increase or a decrease in bacterial growth. Our lowest concentration of each extract in 90% inhibition of bacterial growth was recorded as MIC. The strains were considered sensitive to the control antibiotics if their MIC_90_ values were below 0.002 mg/mL for amoxicillin and below 0.008 mg/mL for metronidazole [48].

#### 2.3.4. Evaluation of Inhibitory Activity of Extracts on Urease

Reaction mixtures comprising of 25 *μ*L of enzyme solution (green bean urease) and 55 *μ*L of buffers containing 100 mM urease were incubated with 5 *μ*L of extracts (concentration of 50 *μ*g/mL) at 30°C for 15 minutes in 96-well plates. Urease activity was determined by measuring ammonia production by the indophenol method as described by the burn of time [50]. Forty five (45) *μ*L of each phenolic reagent (1% w/v phenol and 0.005% w/v sodium nitroprusside) and 70 *μ*L of alkaline reagent (0.5% w/v NaOH and 0.1% chloride active NaOCl) were added to each well. Increase in absorbance at 630 nm was measured after 50 minutes, using a microplate reader (molecular device). All the reactions were carried out in triplicates in a final volume of 200 *μ*L. The results (absorbance change per minute) were processed using softMax Pro software (molecular Device). All the tests were carried out with pH 6.8 [51]. The inhibition percentage was calculated from the formula:

(1) $$\begin{array}{c} \left(\%\right)\text{ Inhibition}=100-\frac{\text{DO test}}{\text{DO control}}\times 100 \end{array}$$

Thiourea has been used as a standard urease inhibitor

### 2.4. Evaluation of the Antioxidant Properties of *A. boonei*

#### 2.4.1. Evaluation of the Antiradical Activity of *A. boonei* by the DPPH (1,1-Diphenyl-2-Picrylhydrazyl) Test

DPPH testing of samples was evaluated as described by Mensor et al. [52]. In each well of a 96-well plate, 20 *μ*L of methanol was introduced in the last seven rows; then, 20 *μ*L of the aqueous or methanol extracts of *A. boonei* (2 mg/mL) was introduced into the first two wells of each column. A volume of 180 *μ*L of methanolic solution of DPPH (0.08 mg/mL) was introduced into each well of the first three columns, while 180 *μ*L of methanol was introduced into each well of the fourth column. Plates containing 200 *μ*L of final solution per well were incubated for 30 minutes in the dark at room temperature; after incubation, optical densities were read on a spectrophotometer (FLUOstar Omega Microplate Reader) at 517 nm and converted into percentages of antioxidant activity. Vitamin C (L-ascorbic acid) was used for positive control. In each sample, three replicates were performed. Percentages of antioxidant activity of each sample were calculated according to the formula

(2) $$\begin{array}{c} \%\text{AA}=\frac{\left[\text{ODDPPH}-\left(\text{ODtest}-\text{ODWhite}\right)\right]}{\text{ODDPPH }}\times 100 \end{array}$$

where *AA* is the antioxidant activity, *OD* is the absorbance, *Test* is the sample + methanolic solution of DPPH, and *Blank* is the sample + methanol

The different percentages of antioxidant activity were used to determine the EC_50_.

#### 2.4.2. Evaluation of the Reducing Power of Extracts by the FRAP (Ferric Reducing Antioxidant Power) Method

The reduction power of the samples was determined according to the protocol prescribed by Benzie and Strain [53]. FRAP reagent was prepared by mixing sodium acetate buffer solution (300 mM, pH 3.6), 2, 4,6-tris (2-pyridyl)-1,3,5-s solution-triazine TPTZ (10 mM), and a solution of FeCl_3_ in the proportions 10 : 1 : 1. A volume of 5 *μ*L of sample (2 mg/mL) was mixed with 95 *μ*L of FRAP reagent. This mixture was incubated for a duration of 30 minutes at 37°C in the dark. After the incubation process, the optical density was read on a spectrophotometer (FLUOstar Omega microplate reader) at 593 nm. Vitamin C was used as a positive control. The antioxidant power of the sample was calculated from the calibration curve of the FeSO_4_ solution and expressed in FeSO_4_ micromole equivalent per gram of sample.

### 2.5. Evaluation of anti-Inflammatory Properties

#### 2.5.1. Evaluation of the Effect of *A. boonei* Extracts on Proteinase

The protease inhibitory activity was carried out using the method of Sakat et al. [54]. Two milliliters of 6% trypsin and 1 mL of tris buffer, HCl (20 Mm; pH: 7.4) were added to 1 mL of each extract (aqueous and methanol) or diclofenac at different concentrations (67.5, 125, 250, 500, and 1000 *μ*g/mL), and this mixture was incubated at 37°C for a duration of 5 minutes, and then, 1 mL of 0.8% casein was added. The mixture was further incubated for another 20 minutes. Two milliliters of perchloric acid (70%) was added to stop the reaction, the cloudy suspension was centrifuged, and the absorbance of the supernatant was read at 120 nm against blank (tris buffer). The percentage inhibition of protease activity was calculated according to the formula

(3) $$\begin{array}{c} \left(\%\right)\text{ Inhibition}=\frac{\text{ODControl}-\text{ODtest}}{\text{ODcontrol}}\times 100 \end{array}$$

#### 2.5.2. Evaluation of the Effect of *A. boonei* Extracts on Protein Denaturation

The inhibitory activity of protein denaturation of the aqueous and methanol extracts of *A. boonei* was carried out according to the method described by Sakat et al. [54]. The reaction mixture (2 mL contained 0.06 mg of trypsin, 1 mL of 20 mM Tris-HCl buffer (pH 7.4), and a sample of *A. boonei* of 1 mL for each extract or diclofenac at different concentrations (0.1, 1, 10, 100, and 1000 *μ*g/mL)) was also incubated at a temperature of 37°C for 5 minutes, and then, 1 mL of casein at (0.8%; m/v) was added. The mixture was further incubated for an additional 20 minutes, and 2 mL of 70% perchloric acid was added to stop the reaction. The cloudy suspension was centrifuged, and the absorbance of the supernatant was read at 210 nm using a URIT 810 spectrophotometer against the white tube. The experiment was carried out in the copies. The inhibition percentage of protein denaturation was calculated using the optical densities (OD) as follows:

(4) $$\begin{array}{c} \left(\%\right)\text{ Inhibition}=\frac{\text{ODcontrol}-\text{DOtest}}{\text{ODcontrol}}\times 100 \end{array}$$

#### 2.5.3. Evaluating the Activity of *A. boonei* Extracts on Nitric Oxide Production

*(1) Isolation and Purification of Peritoneal Macrophages*. The protocol used is that described by Zhang et al. [55]. Thus, mice aged between 8 to 10 weeks, weighing between 20 to 30 g, were sacrificed by cervical dislocation; their abdomens were cleaned and disinfected with 90% ethanol, and then, a small incision was made in the skin to expose the muscles of the abdominal wall. Five milliliters of PBS/EDTA buffer was injected into the peritoneal cavity using a sterile 5-mL syringe with a 25G needle. After massaging the abdomen for about 10-15 seconds, the fluid from the abdominal cavity was collected and dispensed into sterile capped tubes and centrifuged at 1500 rpm for 5 minutes, and the cells were washed with PBS by a second centrifugation. Under a laminar flow hood, the supernatant was removed, and 2 mL of the previously prepared RPMI complete medium was added to resuspend the cell pellet. Ten microliters of this suspension was taken and loaded into the chamber of a Malassez slide; then, the number of cells was determined. After counting, cells were seeded into wells of 96-well plates at 10^5^ cells per well and incubated at 37°C in a 5% CO_2_ and 90% humidity incubator for a duration 3 hours. During this time, macrophages, having the property of attaching to the plastic surface, adhered, and the nonmacrophage cells and the dead cells remained in suspension and were excluded from the medium. The macrophages having adhered were washed with PBS and used for the various tests.

*(2) Exposure of Macrophages to Aqueous Extracts and Methanol from A. boonei*. After counting, the cells were distributed in different wells at 10^5^ cells/mL. In the test and positive control wells, 150 *μ*L of cells was introduced with 50 *μ*L of LPS (1 *μ*g/mL); in the blank wells, 150 *μ*L of cells was introduced with 50 *μ*L of DMEM (Dulbecco's modified Eagle's medium). The microplate was incubated for a period of 1 hour at a temperature of 37°C, then, 50 *μ*L of extracts or diclofenac at different concentrations (0.1, 1, 10, 100 and 1000 *μ*g/mL) was added to the test wells, and 50 *μ*L of DMEM was added to blank and positive control wells. Microplate was again incubated for a period of 3 hours at 37°C. The supernatant was used for the determination of nitric oxide.

#### 2.5.4. Evaluation of the Activity of Extracts on the Stabilization of Erythrocyte Membranes

This test is used to assess the osmotic fragility of erythrocyte cells in the face of thermal insult, as described by Sakat et al. [54].

*(1) Preparation of the Erythrocyte Suspension*. Fresh rat blood was collected and transferred to tubes containing EDTA (ethylene diamine tetra-acetic acid) and then centrifuged at 3000 rpm during 10 minutes at a temperature of 25°C. The supernatant was washed three times. Blood was measured and reconstituted as a suspension (10%; v/v) with physiological saline [54].

*(2) Hemolysis Induced by Heat*. The reaction mixture (2 mL) was composed of 1 mL of aqueous or methanol extracts of *A. boonei* of concentrations (0.1, 1, 10 100, and 1000 *μ*g/mL) and 1 mL of suspension of red blood cells at 10%. Saline solution was added in the control test tube. Diclofenac sodium was used as a reference substance. All the centrifuge tubes, which contained the reaction mixture, were incubated in a water bath at 56°C for a period of 30 minutes. At the end of the incubation process, the centrifuge tubes were cooled under a running tap of water. The reaction mixture was centrifuged at 2500 rpm for a period of 5 minutes, and the absorbance of supernatant was read at 560 nm on a spectrophotometer. The experiment was carried out in triplicate for all the samples that was tested. The membrane percentage stabilizing activity was calculated using the formula [54]

(5) $$\begin{array}{c} \left(\%\right)\text{ Inhibition}=\frac{\text{ODControl}-\text{ODtest}}{\text{ODcontrol}}\times 100 \end{array}$$

### 2.6. Evaluation of the Effect of Extracts on Cell Viability with 3-(4,5-Dimethylthiazol-2-yl)-2,5-Diphenyltetrazolium Bromide (MTT)

The cell pellet from the different incubations in the macrophage exposure assay was taken in 100 *μ*L of MTT solution (0.5 mg/mL in PBS), and the mixture was incubated at 37°C for a duration of an hours 30 minutes, after which the supernatant was removed and 100 *μ*L of acidified isopropanol was added into each tube to dissolve the formazan crystals formed. Finally, absorbance of the blue-violet solution was read at 550 nm on a spectrophotometer relative to the acidified isopropanol solution. Cell viability percentages were calculated using the following formula:

(6) $$\begin{array}{c} \left(\%\right)\text{ Viabilit}\overset{´}{\text{e}}=\frac{\text{ODtest group}}{\text{ODcontrol}}\times 100 \end{array}$$

## 3. Statistical Analyses

The results was expressed as mean ± standard deviation using SPSS software (version 17.0) (Chicago, Illinois, 2009) and Excel. The use of one-way analysis of variance (ANOVA), followed by Turkey's post-test, was also used to compare differences between the means of inhibitory activities of extracts and antibiotics. The differences were significant at *p* < 0.05.

## 4. Results

### 4.1. Qualitative and Quantitative Phytochemistry

The qualitative analysis of the barks of the trunk of *A. boonei* showed that the two extracts contain 6 classes of secondary metabolites, namely, alkaloids, flavonoids, tannins, polyphenols, triterpenoids, and saponins. In addition to the 6 metabolites, the methanol extract contains 2 other metabolites (anthocyanins and anthraquinones) which are absent in the aqueous extract. In addition, we note the absence of glycosides and sterols in the two extracts (Table 1).

All concentration sites of the compounds found in the two extracts are shown in Table 2. The concentrations of flavonoids, total polyphenols, and tannins were higher in the methanol extract (126.70 mg quercetin/mg), (258.00 mg of catechin/mg of extract), and (155.60 mg of tannic acid/mg of extract), respectively, compared to the aqueous extract (99.28 mg of quercetin/mg), (244.40 mg of catechin/mg extract), and (132.80 mg tannic acid/mg aqueous extract), respectively. All compounds were higher in methanol extract compared to that of aqueous extract.

### 4.2. Effect of Extracts on some *H. pylori* Activity

#### 4.2.1. Effects of Aqueous and Methanol Extracts of *A. boonei* on some Strains of *H. pylori*

The activity of the two extracts on the 15 isolates of *H. pylori* made it possible to obtain the inhibition zone diameters which ranged from 7 to 35 mm for the aqueous extract and from 7 to 36 mm for the methanol extract (Table 3). Maximal inhibition was obtained with the methanol extract for an inhibition zone diameter range of 7-36 mm compared to clarithromycin and the aqueous extract. Both extracts had recorded a larger mean zone diameter of 16.88 mm and 15.35 mm for the methanol extract and the aqueous extract, respectively, compared to clarithromycin (12.98 mm).

**Figure 1:**
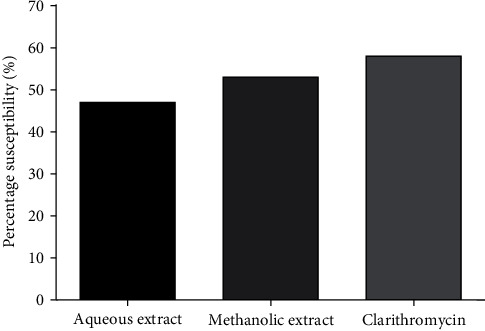
Effects of aqueous and methanol extracts of *A. boonei* on *H. pylori* by the BHI (Brain Heart Infusion) gelose well diffusion method.

Figure 1 shows that the isolates were sensitive for both extracts and clarithromycin. This figure presents a percentage of sensitivity (< 50%) of *H. pylori* isolates for the aqueous extract; on the one hand, for methanol extract and clarithromycin, the percentage of sensitivity is >50%. Clarithromycin had a higher percentage of sensitivity (58%) compared to the methanol extract and the aqueous extract, *i.e.*, 58% and 48%, respectively. Of the two extracts, the methanol extract had shown a higher sensitivity percentage (55%) than the aqueous extract (48%). It is noted that the aqueous extract has the lowest percentage of sensitivity to *H. pylori* isolates.

*A. boonei* extracts had shown differences in anti-*H. pylori* activity from each other with MIC values which ranged between 1.3 and 7.75 mg/mL (Table 4). Methanol extract of *A. boonei* compared to the aqueous extract had shown the best activity for MIC_90_ values between 1.3 and 4.45 mg/mL, on the one hand, while aqueous extract gave MICs ranging from 3.5 to 9 mg/mL. The MIC_90_ values of amoxicillin and metronidazole were between 0.001 and 6 mg/mL. Among the two extracts, it appears that the methanol extract had shown a minimum inhibitory concentration (MIC_90_) lower (2.84 mg/mL) than those of the aqueous extract (6.58 mg/mL) and metronidazole (3.40 mg/mL) and better than that of amoxicillin (0.50 mg/mL). The MIC_90_ of the methanol extract is between that of metronidazole and that of amoxicillin.

#### 4.2.2. Effects of Aqueous and Methanol Extracts of *A. boonei* on Urease

**Figure 2:**
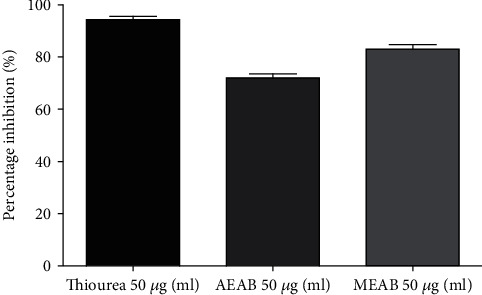
Antiureasic effect of aqueous and methanol extracts of *A. boonei*.

Antiurease effects of *A. boonei* extracts are summarized in Figure 2. It appears that aqueous and methanol extracts, such as thiourea, had shown significant antiurease activity. Indeed, the percentages of inhibitions obtained for the aqueous and methanol extracts and the thiourea were, respectively, 73%, 85%, and 94%. Of the two extracts, the methanol extract has higher percentages of inhibition (85%) compared to the aqueous extract (73%). Thiourea had the greatest percentage of inhibition, 94%.

### 4.3. Antioxidant Properties of Aqueous and Methanol Extracts of *A. boonei*

#### 4.3.1. Effects of Aqueous and Methanol Extracts of *A. boonei* on DPPH Radical Scavenging

**Figure 3:**
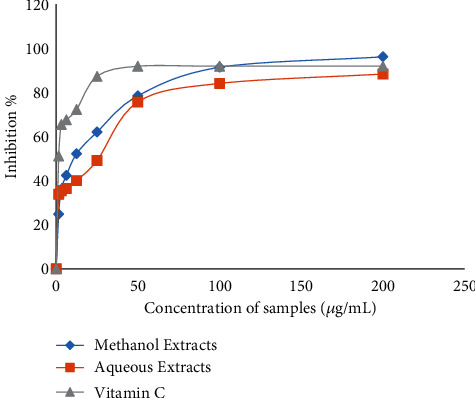
Effects of aqueous and methanol extracts of *A. boonei* on DPPH radical trapping.

Figure 3 and Table 5 show the EC_50_ values of aqueous and methanol extracts and vitamin C of *A. boonei.* The EC_50_ value obtained with vitamin C was the lowest (2.29 *μ*g/mL), followed by that of the methanol extract (8.91 *μ*g/mL) and finally the aqueous extract with 19.86 mcg/mL. The EC_50_ value of the methanol extract (8.91 *μ*g/mL) was very low compared to that of aqueous extract (19.86 *μ*g/mL). Concentration at 200 *μ*g/mL of methanol extract had shown a higher percentage of inhibition (96.34%) than that of the aqueous extract (88.52%). Vitamin C had for its part shown a trapping power of 92.06% at this same concentration (200 *μ*g/mL) higher than that of the aqueous extract and lower than that of methanol extract. Vitamin C had also presented a percentage inhibition of (92.06%) greater than the various extracts at the concentration of 50 *μ*g/mL. When concentration is at 100 *μ*g/mL, vitamin C (92.06%) and the methanol extract (91.57%) had presented an inhibition percentage almost identical and superior to that of the aqueous extract (84.25%).

#### 4.3.2. Effects of Aqueous and Methanol Extracts of *A. boonei* on the Reducing Power of Iron (FRAP)

The iron-reducing power of the aqueous and methanol extract of the sterm bark of the trunk of *A. boonei* is presented in Table 6. The absorbance of the different samples increased with the concentration. The methanol extract (108.8 mmol FeSO_4_/g) exhibited higher iron reducing activity compared to the aqueous extract (92.27 mmol FeSO_4_/g) and vitamin C (60.52 mmol FeSO_4_/g).

### 4.4. Anti-Inflammatory Properties of Aqueous and Methanol Extracts of *A. boonei*

#### 4.4.1. Effects of *A. boonei* Extracts on Proteinase Activity

The antiproteinase effects of the aqueous and methanol extracts of *A. boonei* are summarized at Table 7. This table shows that at concentrations 67.5, 125, and 250 *μ*g/mL, the percentages of inhibition of the aqueous extract 42.79%, 57.13%, and 62.89%, respectively, were higher than those of the methanol extract 29.93%, 41.81%, and 58.72%, respectively. Both extracts, including diclofenac, had shown significant antiproteinase activity with percentage inhibitions ranging from 71.43%, 77.33%, and 96.10%, respectively, at the concentration of 1000 *μ*g/mL. The percentage inhibitions are concentration-dependent for the concentrations 500 and 1000 *μ*g/mL in the extracts as in diclofenac. Diclofenac showed greater inhibition percentages than both extracts for all concentrations, except for the concentration of 67.5 *μ*g/mL were its percentage inhibition (41.02%) is lower than that of the aqueous extract (42.02%) and higher than that of the methanol extract (29.93%). Of the two extracts, the methanol extract was the one that presented the greatest percentage of inhibition (77.33%) compared to the aqueous extract (71.43%) at the concentration of 1000 *μ*g/mL.

#### 4.4.2. Effects of *A. boonei* Extracts on Protein Denaturation

Table 8 shows the effect of aqueous and methanol extracts of *A. boonei* on protein denaturation. From this table, it is apparent that aqueous and methanol extracts of *A. boonei* at all concentrations had inhibited protein denaturation in a concentration-dependent manner. The percentage inhibition of the methanol extract was greater than that of the aqueous extract and of diclofenac at all concentrations and the percentage inhibition of diclofenac was greater than that of the aqueous extract concentration of 1000 *μ*g/mL. The maximum percentage of inhibition was obtained with the methanol extract (84.98%) compared to diclofenac (82.19%) and the aqueous extract (73.44%).

#### 4.4.3. Effects of Extracts of *A. boonei* on the Stabilization of Erythrocyte Membranes

Both *A. boonei* extracts inhibited heat-induced red blood cell hemolysis at all concentrations (Table 9). This table shows that the percentage inhibitions are concentration-dependent; at all concentrations, the percentage inhibition of diclofenac was greater than that of the methanol extract and the aqueous extract; and the percentage inhibition of the methanol extract is higher than that of the aqueous extract. Diclofenac showed a maximum percentage inhibition (94.78%) compared to the methanol extract (76.37%) and the aqueous extract (59.70%) at the concentration of 1000 *μ*g/mL. The extract that showed a better percentage of inhibition of membrane hemolysis was the methanol extract compared to the aqueous extract at all concentrations.

#### 4.4.4. Effects of *A. boonei* Extracts on the Production of Nitric Oxide (NO)

Table 10 shows that diclofenac had not induce any modification in the production of NO by macrophages, whether cells with Saccharomyces or not. Similarly, it is noted that the two extracts did not affect cell viability and the production of NO by macrophages in cells without Saccharomyces. On the other hand, in the cells placed in the presence of Saccharomyces, a strong production of NO is noted when these were previously treated with extracts of *A. boonei*. This increase is observed at all concentrations of the two extracts. The concentrations 10, 100, and 1000 *μ*g/mL for the methanol extract present low concentrations of nitrite and tend to decrease the production of NO.

### 4.5. Effects of *A. boonei* Extracts on Cell Viability

The results in Table 11 show that at the highest concentration (1000 *μ*g/mL), we observe that the cells are more than 92.04% viable for the aqueous extract and more than 83.65% for the methanol extract. The aqueous extract compared to the methanol extract presented the greatest percentage of inhibition at all concentrations although these percentages were not nearly similar; those of the aqueous extract were higher than those of the methanol extract. These results suggest that aqueous and methanol extracts at all concentrations do not have cytotoxic activity. These concentrations were chosen for the tests on the anti-inflammatory activities.

## 5. Discussion

As part of this work, the antioxidant activities (FRAP and DPPH tests), the anti-*H. pylori* (determination of MIC_90_, urease and protease enzyme inhibition tests), and anti-inflammatory (protein denaturation, production of NO by macrophages, cytotoxicity, and hemolysis of red blood cells by heat) were carried out.

*H. pylori* is a Gram-negative microaerophilic bacterium, which colonizes the stomach mucus affects half of the world's population. All individuals infected with *H. pylori* present microscopic gastritis characterized by the infiltration of chronic inflammatory cells, with an accumulation of neutrophil leukocytes [14]. *H. pylori* is the only bacterial species known to be carcinogenic to humans [56]; it produces substances that degrade mucin and injure epithelial cells, thereby reducing mucosal resistance to acid damage. *H. pylori* infection works by perforating the stomach with its flagella; once adhered to the gastric mucosa, *H. pylori* will inject its virulent factors, in particular the cagA protein, into the host's epithelial cells through the type IV secretion system. The in vitro results of this study showed maximum inhibition obtained with the methanol extract for a diameter range of the inhibition zone ranging from 7 to 36 mm. Aqueous and methanol extracts of *A. boonei* produced a larger mean zone diameter of 16.88 mm and 15.35 mm, respectively, compared to clarithromycin (12.98 mm). Similarly, the aqueous extract showed a percentage sensitivity of less than 50%, while the methanol extract and clarithromycin showed percentages of sensitivity greater than 50%. The methanol extract showed a lower minimum inhibitory concentration (MIC90) than the aqueous extract and metronidazole and better than that of amoxicillin. These results are justified by the presence in the extracts of flavonoids, phenolic compounds, and alkaloids since these classes of compound have already shown numerous anti-*H. pylori* [57–59].

It is known that whenever ure is absent, the body's metabolic pH range is that of a neutralophile, so once in the human body, *H. pylori* must maintain its metabolism and its synthesis of ATP in higher acidity than in organisms unable to grow in the stomach, for this constitutive urease production is a major component, although other mechanisms may also play a role [60]. Urease is an important pathogenic factor which helps *H. pylori* in colonizing the epithelium in the acid environment of the stomach [61]. This enzyme exists in two forms: cytoplasmic and surface proteins which release NH3 by hydrolyzing urea [62]. A rapid rise in environmental pH by urease activity allows the survival of the microorganism in acidic environments. Thus, the urease-dependent elevation of local pH to nontoxic levels allows for the survival of *H. pylori* [63–65]. *H. pylori* maintains its periplasmic pH even when it is exposed to media which is relatively high in acidity. Urease activity protects the body in the absence of raising the pH of the medium to nontoxic levels, so it is the body's internal urease that is protective [66]; the internal activity of urease under acidic conditions must first of all elevate the periplasmic, not the mean, pH to allow its protective action. Urease activity is the main reason why *H. pylori* is an acid-tolerant neutralophile [60]. As the activity of urease seems crucial for the survival and the transmission of the bacterium, we evaluated in this study the effects of our extracts on the activity of urease in vitro, and it appears that the two extracts have showed significant antiureasic activities with inhibition percentages of 73% and 85%, respectively, for the aqueous and methanolic extracts. It has been demonstrated that the inhibitory effect of certain flavonoids against *H. pylori* is due to the inhibition of urease activity [67], which may justify the activity of extracts of *A. boonei*, since qualitative and quantitative phytochemical tests confirm the presence of these class of compounds within the various extracts of *A. boonei*.

*H. pylori* infection is a causative factor in various disorders of the gastric epithelium, including ulceration, metaplasia, dysplasia and carcinoma [68]. It is known that the increase levels of reactive oxygen are generated in gastric epithelial cells infected with *H. pylori* and this might be a mechanism which leads to infections associated with apoptosis [68]. Furthermore, antioxidants prevent the generation of ROS and inhibit programmed cell death induced by *H. pylori* for the prevention and treatment of these common and chronic infectious diseases. Superoxide anion was detected in epithelial cells preparations isolated from guinea pig gastric mucosa after experimental *H. pylori* infection [69]. *H. pylori* infection stimulates the accumulation of intracellular ROS (reactive oxygen species) in different human gastric epithelial cell lines [70, 71]. Reports demonstrating decreased levels of GSH in human gastric epithelial cells infected with *H. pylori* [72, 73] further provide evidence that *H. pylori* serves as a stimulus for the accumulation of ROS in gastric epithelial cells. People infected with *H. pylori*, their levels of nitric oxide, and reduced glutathione are lower, while the levels of malondialdehyde, catalase, and superoxide dismutase are higher [74]. Our results showed that the extracts of *A. boonei* have antiradical properties (DPPH) and also have iron-reducing power, which confirm the antioxidant properties of these plant extracts. It is known that many compounds have shown *in vitro* antiradical properties and the reducing power of iron has antioxidant properties.

*H. pylori* infection has been associated with the generation of reactive oxygen species (ROS) that leads to oxidative stress therein the gastric mucosa [75, 76]. This bacterium induces the infiltration and activation of phagocytes, which produces inflammatory mediators, cytokines, and ROS. *H. pylori* activates inducible nitric oxide synthase within the gastric mucosa, which is associated with epithelial cell damage and apoptosis [77]. NO and reactive oxygen species affect virtually every stage in the development of inflammation, in particular, plants that reduce NO formation might be beneficial in pathophysiological conditions where excess NO production is a contributing factor agent that reduces NO formation which may be beneficial in *H. pylori* infections. This is so because the excess NO production is an aggravating factor in this condition. Finally, increased production of free radicals has been shown to occur during the gastrointestinal metabolism of xenobiotics, leading to intestinal disorders [78]. Based on these results, *A. boonei* is considered as a source of compounds with anti-*H. pylori*, but it should be used with caution in the treatment of gastritis and peptic ulcers, since reactive oxygen/nitrogen intermediates are implicated in the pathogenesis of ulcerogenic agents induced by gastric mucosal damage and gastric infections of *H. pylori.*

Activated neutrophils or macrophages during *H. pylori* infection are known to be sources of ROS [79]. In addition, cytokines increased in gastric mucosa of infected persons [80] can also induce oxidative stress and increased oxidative responses to *H. pylori*; cytokine-mediated oxidative signaling occurs in gastric epithelial cells. *H. pylori* infection has proven to be associated with increased gastric inflammation, increased bacterial load, and both peptic ulcer disease and gastric cancer [81, 82]; this infection induces high levels of IL-8 [83] and activates the transcription factors NF-*κ*B (*nuclear factor-κapaB*) [84] and AP-1 (activator protein 1) [85]. *H. pylori* colonizes the human gastric epithelium, living in the mucus layer in close proximity to epithelial surfaces, without invading the mucosa. There are two main mechanisms by which *H. pylori* can produce gastric inflammation. In the first place, organisms can interact with surface epithelial cells, producing either direct cellular damage or the release of epithelium-derived proinflammatory mediators (chemokines). In the second place, *H. pylori*-derived products can access underlying mucosa, directly stimulating nonspecific and host-specific immune responses involving the release of a variety of cytokine messengers [86]. Surface epithelial degeneration correlates with the number of *H. pylori* in close contact with the epithelial plasma membrane, a finding that supports a direct toxic effect of bacterial products on epithelial cells [87]. About 50% of *H. pylori* strains produce a heat-labile, protease-sensitive vacuolating cytotoxin that induces vacuole formation in cultured epithelial cells [88, 89]. Mononuclear phagocytes play a central role in early immune responses to bacteria, serving as an important source of proinflammatory mediators and antigen-presenting cells involved in the initiation of specific immunity. Soluble proteins derived from *H. pylori* can activate peripheral blood monocytes resulting in increased expression of inflammatory cytokine and tumor necrosis factor production and superoxide anion secretion [90]. The balance between pro-inflammatory and immunosuppressive cytokines is a critical determinant of the severity of inflammations associated with *H. pylori* [91]. Aqueous and methanol extracts of *A. boonei*, including diclofenac, demonstrated significant antiproteinase activity with percentage inhibitions ranging from 71.43%, 77.33%, to 96.10%, respectively, at the concentration of 1000 *μ*g/mL. Extracts at all concentrations inhibited protein denaturation in a concentration-dependent manner. Both extracts of *A. boonei* extracts inhibited heat-induced red blood cell hemolysis at all concentrations. These results confirm the anti-inflammatory properties of aqueous and methanol extracts of *A. boonei.* Certain flavonoids inhibit the activation of NF-*κ*B and thus reduce the expression of inflammatory factors; in addition to other natural polyphenols, suppresses the activation of NF-*κ*B as well as the activation of IKK and the degradation of I*κ*B*α* and thus the inflammatory process of cells infected by *H. pylori* [92]. Terpenoids inhibit *H. pylori*-induced IL-8 production by inhibiting IKK and NF-*κ*B activation in a dose- and in a time-dependent manner [93]. The activities of the different extracts observed in this study would therefore be linked to the presence of flavonoids, polyphenols, and terpenoids within the different extracts, without ruling out the possibility of a synergistic effect.

## 6. Conclusion

It appears from this study that aqueous and methanol extracts of *A. boonei* possess anti-*H. pylori* by inhibiting bacterial activity and inhibiting urease activity. These extracts also have antioxidant properties through antiradical activity and reducing power and then anti-inflammatory activities through inhibition of proteinase, protein denaturation, and nitric oxide production. The activities of this plant would be linked to the presence of flavonoids, phenolic compounds, and terpenoids. This study confirms the traditional use of *Alstonia boonei* (Apocynaceae) in the treatment of gastric pathologies and proves that this plant is a source of new secondary metabolites for the management of gastric pathologies in general and gastric pathologies related to *H. pylori* in particular.

## Figures and Tables

**Table 1:** Qualitative phytochemical screening of aqueous and methanol extracts of de *A. boonei.*

Compounds and extracts	Aqueous extract	Methanol extract
Alkaloids	+	+
Flavonoids	+	+
Tannins	+	+
Polyphenols	+	+
Triterpenes	+	+
Sterols	—	—
Saponins	+	+
Glycosides	—	—
Anthocyanins	—	+
Anthraquinones	—	+

+: present; -: absent.

**Table 2:** Quantitative phytochemical screening of aqueous and methanol extracts of *A. boonei.*

	Flavonoids (mg/g E quercetin)	Polyphenols (mg/g E catechin)	Tannins (mg/g E tannic acid)
Aqueous extract	99.28 ± 1.33	244.40 ± 1.12	132.80 ± 1.77
Methanol extract	126.70 ± 0.89	258.00 ± 8.08	155.60 ± 8.48

The levels of flavonoids, total polyphenols, and tannins are expressed as milligram equivalents of quercetin, milligram equivalents of catechin, and milligram equivalents of tannic acid, respectively.

**Table 3:** Screening of aqueous and methanol extracts of *A. boonei* against *H. pylori* isolates.

	Mean zone diameter (mm)	Inhibition zone diameter range (mm)
Clarithromycin	12.98 ± 4.87	0–31
Aqueous extract	15.35 ± 5.33	7–35
Methanol extract	16.88 ± 3.45	7–36

Data are mean values ± standard deviation of 16 independent determinations for each extract or antibiotic control.

**Table 4:** Minimum inhibitory concentration (90%) of aqueous and methanol extracts of *A. boonei* (mg/mL).

	MIC_90_ (mg/mL)			
Strains *H. pylori*	AE	ME	Metronidazole	Amoxicillin
1	3.5	2.26	2.6	0.04
2	5.5	2.1	—	0.02
3	5	1.3	—	0.99
4	6.8	3.55	—	0.91
5	7.5	3.6	—	0.02
6	6.5	4.45	3	0.03
7	4.4	2.45	5	0.05
8	8	3.31	—	0.07
9	9	2.2	—	0.1
10	7.5	2.5	1.9	0.09
11	8.5	3.45	3	1
12	7.9	2.7	3.5	1.2
13	8	2.55	5	1.4
14	7	2.5	6	2
15	7.75	4	4	0.04
NCTC11638	2.5	2,5	0.004	0.001
Mean ± SD	6.58 ± 0.47	2.84 ± 0.20	3.40 ± 0.54	0.50 ± 0.16

**Table 5:** Effects of aqueous and methanol extracts of *A. boonei* on DPPH radical trapping.

Samples	CE_50_ (*μ*g/mL)
Aqueous extract	19.86 ± 0.65
Methanol extract	8.91 ± 0.26
Vitamin C	2.29 ± 0.13

**Table 6:** Effects of aqueous and methanol extracts of *A. boonei* on the reducing power of iron.

Samples	FRAP assay (mmol FeSO_4_/g)
Aqueous extract	92.27 ± 0.42
Methanol extract	108.80 ± 0.75
Vitamin C	60.52 ± 0.35

**Table 7:** Effect of aqueous and methanol extracts of *A. boonei* on proteinase.

	Concentration (*μ*g/mL)	Inhibition (%)
Aqueous extracts	67.5	42.79 ± 2.85
	125	57.13 ± 2.61
	250	62.89 ± 4.25
	500	71.80 ± 1.16
	1000	71.43 ± 0.43

Methanol extracts	67.5	29.93 ± 1.20
	125	41.81 ± 1.76
	250	58.72 ± 0.54
	500	75.42 ± 3.84
	1000	77.33 ± 0.33

Diclofenac	67.5	41.02 ± 3.27
	125	65.84 ± 3.28
	250	83.97 ± 0.71
	500	90.30 ± 3.35
	1000	96.10 ± 0.43

**Table 8:** Effect of aqueous and methanol extracts of *A. boonei* of proteins denaturation.

	Concentration (*μ*g/mL)	Inhibition (%)
Aqueous extracts	0.1	22.81 ± 0.47
	1	38.59 ± 0.33
	10	53.12 ± 0.76
	100	62.12 ± 0.30
	1000	73.44 ± 0.35

Methanol extracts	0.1	39.72 ± 1.39
	1	56.47 ± 3.21
	10	67.13 ± 3.11
	100	75.74 ± 5.02
	1000	84.98 ± 1.93

Diclofenac	0.1	32.18 ± 0.61
	1	41.29 ± 2.94
	10	65.02 ± 0.80
	100	71.41 ± 0.41
	1000	82.19 ± 4.21

**Table 9:** Effects of aqueous and methanol extracts of *A. boonei* on the stabilization of erythrocyte membranes.

	Concentration (*μ*g/mL)	Inhibition (%)
Aqueous extracts	0.1	16.17 ± 3.89
	1	21.64 ± 3.37
	10	43.53 ± 4.32
	100	51.99 ± 0.50
	1000	59.70 ± 1.55

Methanol extracts	0.1	33.83 ± 0.66
	1	46, 77 ± 0.25
	10	52.24 ± 1.97
	100	62.69 ± 0.86
	1000	76.37 ± 1.79

Diclofenac	0.1	45.02 ± 2.52
	1	66.67 ± 1.74
	10	75.37 ± 1.49
	100	82.59 ± 1.74
	1000	94.78 ± 0.86

**Table 10:** Effect of aqueous and methanol extracts of *A. boonei* on NO production by macrophages.

	Concentration (*μ*g/mL)	Macrophages (with saccharomyces) (*μ*g/mL)	Macrophages (without saccharomyces) (*μ*g/mL)
Aqueous extracts	0.1	3.56 ± 0.10	0.15 ± 0.04^c^
	1	2.94 ± 0.12	0.36 ± 0.02^c^
	10	2.07 ± 0.30^b^	0.41 ± 0.06^c^
	100	2.47 ± 0.09	0.59 ± 0.03^c^
	1000	2.64 ± 0.12	1.34 ± 0.02^c^

Methanol extracts	0.1	2.89 ± 0.14	0.13 ± 0.01^c^
	1	2.51 ± 0.11^a^	0.22 ± 0.04^c^
	10	1.94 ± 0.11^c^	0.20 ± 0.02^c^
	100	1.51 ± 0.07^c^	0.28 ± 0.08^c^
	1000	1.65 ± 0.09^c^	1.16 ± 0.06^c^

**Table 11:** Effect of aqueous and methanol extracts of *A. boonei* on cell viability.

	Concentration (*μ*g/mL)	Inhibition (%)
Aqueous extracts	0.1	104.10 ± 3.44
	1	100.70 ± 1.65
	10	102.10 ± 1.14
	100	95.43 ± 1.47
	1000	92.04 ± 0.27

Methanol extracts	0.1	101.20 ± 3.77
	1	91.52 ± 2.95
	10	100.20 ± 4.59
	100	88.65 ± 0.63
	1000	83.65 ± 0.63

## Data Availability

The datasets used and/or analyzed during the current study are available from the corresponding author on reasonable request.

## Abbreviations

NO: Nitrite oxide
FRAP: Ferric reducing antioxidant power
DPPH: 2,2-Diphenyl-1-picrylhydrazyl
EC50: Median effective concentration
*MIC90*: Minimum inhibitory concentration
DNA: Deoxyribonucleic acid
GAE: Gallic acid equivalents
NaNO2: Sodium nitrite
AlCl3: Aluminum chloride
NaOH: Sodium hydroxide
PVPP: Polyvinylpolypyrrolidone
ICCBS: International Center for Chemical and Biological Sciences
BHI: Brain heart infusion
ELISA: Enzyme-linked Immuno assay
*DMEM*: Dulbecco's modified Eagle medium
EDTA: Ethylene diamine tetra-acetic acid
ROS: Reactive oxygen species
*NF*-*κB*: *Nuclear factor*-*κB*.
